# Hybrid Nanoparticles as an Efficient Porphyrin Delivery System for Cancer Cells to Enhance Photodynamic Therapy

**DOI:** 10.3389/fbioe.2021.679128

**Published:** 2021-09-17

**Authors:** Letícia B. Silva, Kelly A. D. F. Castro, Caroline E. A. Botteon, Cristiano L. P. Oliveira, Roberto S. da Silva, Priscyla D. Marcato

**Affiliations:** ^1^Department of Pharmaceutical Science, GNanoBio, School of Pharmaceutical Sciences of Ribeirão Preto, University of São Paulo, Ribeirão Preto, Brazil; ^2^Department of Biomolecular Sciences, School of Pharmaceutical Sciences of Ribeirão Preto, University of São Paulo, Ribeirão Preto, Brazil; ^3^Institute of Physics, University of São Paulo, São Paulo, Brazil

**Keywords:** hybrid nanoparticles, chitosan, porphyrin, PDT-photodynamic therapy, bladder cancer cells

## Abstract

Photodynamic therapy (PDT) is a potential non-invasive approach for application in oncological diseases, based on the activation of a photosensitizer (PS) by light at a specific wavelength in the presence of molecular oxygen to produce reactive oxygen species (ROS) that trigger the death tumor cells. In this context, porphyrins are interesting PS because they are robust, have high chemical, photo, thermal, and oxidative stability, and can generate singlet oxygen (^1^O_2_). However, porphyrins exhibit low solubility and a strong tendency to aggregate in a biological environment which limits their clinical application. To overcome these challenges, we developed hybrid nanostructures to immobilize 5,10,15,20-tetrakis[(4-carboxyphenyl) thio-2,3,5,6-tetrafluorophenyl] (**P**), a new third-generation PS. The biological effect of this system was evaluated against bladder cancer (BC) cells with or without light exposition. The nanostructure composed of lipid carriers coated by porphyrin-chitosan (**P-HNP**), presented a size of *ca.* 130 nm and low polydispersity (*ca.* 0.25). The presence of the porphyrin-chitosan (**P**-chitosan) on lipid nanoparticle surfaces increased the nanoparticle size, changed the zeta potential to positive, decreased the recrystallization index, and increased the thermal stability of nanoparticles. Furthermore, P-chitosan incorporation on nanoparticles increased the stability and enhanced the self-organization of the system and the formation of spherical structures, as observed by small-angle X-ray scattering (SAXS) analysis. Furthermore, the immobilization process maintained the **P** photoactivity and improved the photophysical properties of PS, minimizing its aggregation in the cell culture medium. In the photoinduction assays, the **P-HNP** displayed high phototoxicity with IC_50_ 3.2-folds lower than free porphyrin. This higher cytotoxic effect can be correlated to the high cellular uptake of porphyrin immobilized, as observed by confocal images. Moreover, the coated nanoparticles showed mucoadhesive properties interesting to its application *in vivo*. Therefore, the physical and chemical properties of nanoparticles may be relevant to improve the porphyrin photodynamic activity in BC cells.

## Introduction

Photodynamic therapy (PDT) is a non-invasive approach to treat oncological and non-oncological diseases (Figueira et al., 2014; Kwiatkowski et al., 2018; Mesquita et al., 2018a,b; Gazzi et al., 2019; Negri et al., 2019; Castro et al., 2020; Gomes et al., 2020; Lee et al., 2020). PDT is a clinically approved treatment based on the activation of a photosensitizer (PS) by the light of a specific wavelength, in the presence of molecular oxygen to produce reactive oxygen species (ROS), resulting in neoplastic cell death (Agostinis et al., 2011; Bogoeva et al., 2016). When the PS is activated, its molecules absorb photons, transmitting energy to molecular oxygen and triggering the production of singlet oxygen (^1^O_2_) (type II), such as other ROS production by an electron transfer mechanism (type I) (Dougherty, 2002; Castano et al., 2004; Purushothaman et al., 2019). These ROS may activate a complex cascade of biochemical and physiological reactions able to induce tumor cell death by apoptosis, necrosis, or autophagy. The damage of microvessels suppresses the malignant tissue nutrition, oxygenation, and promotes adaptive antitumor immunity activation (Garg et al., 2010; Agostinis et al., 2011; Bacellar et al., 2015; Luiza Andreazza et al., 2016).

Bladder cancer (BC) is the tenth highest occurring malignant disease worldwide and the sixth with more incidence in men (Ferlay et al., 2019). In 2018, about 550,000 new cases were reported worldwide (Ferlay et al., 2019). Moreover, 75% of BC cases are diagnosed as non-muscle invasive bladder cancer (NMIBC) or superficial (Tse et al., 2019; Ramuta et al., 2020). Thus, due to the easy access to the bladder and the high exposition of malignant tissue on the bladder surface in cases of NMIBC, PDT has been suggested to enhance the effectiveness of BC therapy (Agostinis et al., 2011; Gomes et al., 2020).

Several porphyrins and their reduced derivatives (chlorins) have been developed and used in the clinic and clinical trials for cancer treatment (e.g., Photofrin or 5-ALA) (Dougherty, 2002; Master et al., 2013; Li et al., 2015; Zhang et al., 2016; Kou et al., 2017; dos Santos et al., 2019).

Porphyrins are aromatic heterocycle compounds formed by four pyrrole rings linked by four methyl bridges. These compounds are robust and have a high chemical, photo, thermal, and oxidative stability (Ptaszyńska et al., 2018). Porphyrins with varied structures and characteristics can be isolated from nature or synthesized. The ability of these derivatives to generate singlet oxygen enables its use as a photosensitizer in PDT (Plaetzer et al., 2003; Allison and Moghissi, 2013; Kou et al., 2017).

Although the use of porphyrin has been proposed as PS used in PDT, the disadvantages of this compound class have been observed, as it has clinical limitations due to its aggregation process (Nawalany et al., 2009; Liang et al., 2014). It is well established in literature that the photosensitizer must maintain a monomeric form to be photoactive (Ricchelli et al., 1998; Chen et al., 2005; Nawalany et al., 2009; Rabiee et al., 2020). Furthermore, several of these photosensitizers have shown low selectivity by cancer tissues (Purushothaman et al., 2019), resulting in the deactivation or reduction of the photosensitizer action. To overcome these challenges, several nanostructures have been developed as a photosensitizer delivery system to increase their selectivity (Zhou et al., 2016; Battogtokh and Ko, 2017; Ujiie et al., 2019).

Berndt-Paetz et al. (2019) reported the success of PDT using tetrahydroporphyrin-tetratosylat (THPTS) encapsulated into liposomes. This system was able to inhibit BC cell growth, trigger cells to apoptosis or necrosis, mainly due to their subcellular localization through the cytoplasm and in lysosomes (Berndt-Paetz et al., 2019). Similar results were reported by Gomes et al. (2020) using polyvinylpyrrolidone micelles containing a triazole-porphyrin derivative.

Mucoadhesive nanoparticles and lipid nanostructures have also been used as PS delivery systems to enhance PDT. The porphyrin 5,10,15,20-tetrakis(*m*-hydroxyphenyl) (*m*THPP) was encapsulated in PLGA nanoparticles coated with poly(ethylene glycol) (PEG) or chitosan (Anderski et al., 2018; Mahlert et al., 2019). These systems showed an increase in the intracellular accumulation of *m*THPP in HT-29-MTX and Caco-2 cells. Moreover, PEG-PLGA-*m*THPP nanoparticles showed superior cytotoxicity to chitosan-PLGA-*m*THPP nanoparticles. The PS chlorin e6 (Ce6) (Lee et al., 2011) and protoporphyrin IX (Lee et al., 2009) conjugated with chitosan nanoparticles and exhibited efficient accumulation in the tumor, exhibiting a superior therapeutic efficacy to free PS in tumor-bearing mice models. The encapsulation of verteporfin in nanostructured lipid carriers (NLCs-verteporfin) increased the uptake of this PS in 2D and 3D models of ovarian cancer cells and triggered a higher phototoxicity effect compared with free verteporfin (Michy et al., 2019). An enhancement of the phototoxic effect of *meso*-(tetra hydroxyphenyl) chlorin (*m*THPC) in breast cancer cells was also obtained when this porphyrin was encapsulated in solid lipid nanoparticles (Navarro et al., 2014). Zhang et al. (2019) developed a NLC with a surface modified with folate to co-delivery Ce6 and paclitaxel. This nanostructured system enhanced the Ce6 and PTX internalization in cancer cells and showed, in an animal model, a significant decrease in the tumor volume compared with groups in dark conditions (Zhang et al., 2019).

Another strategy explored is the use of hybrid nanoparticles. Hybrid nanosystems can be obtained by the combination of different materials (organic–inorganic or organic–organic), combining the attributes of each material to obtain a nanostructure with unique properties (Bochicchio et al., 2018; Mukherjee et al., 2019; Ferreira Soares et al., 2020). Hybrid chitosan-coated gold nanoparticle conjugated with *meso*-tetrakis(4-sulphonatophenyl) porphyrin (TPPS) exhibited high photothermal conversion efficiency, enhancing the cellular uptake of TPPS in HepG2 cells, and showed superior cytotoxicity to free TPPS (Zeng et al., 2018). Thus, the association of PS with nanostructures can improve the photodynamic action, reduce its side effects in healthy tissue (Battogtokh and Ko, 2017; Kwiatkowski et al., 2018; Ujiie et al., 2019), improve its stability and tissue penetration, and enhance the PS uptake in cancer cells (Ujiie et al., 2019).

This study reports on the immobilization of the new 5,10,15,20-tetrakis[(4-carboxyphenyl) thio-2,3,5,6-tetrafluorophenyl] porphyrin (**P**) (Supplementary Figure S1) in a biodegradable hybrid nanoparticle of the NLCs coated with chitosan, aiming to minimize the aggregation process, enhance cellular uptake, and consequently improve the efficiency of this PS in PDT against BC cells.

## Experimental Section

### Materials

In general, the materials were purchased from Sigma-Aldrich without further purification. The Crodamol SS was obtained from CRODA and the *N*,*N*-Dimethylformamide (DMF) from Exodo.

### Porphyrin Synthesis and Quantum Yields of Singlet Oxygen Determination

The porphyrin derivative was prepared according to the literature, following two steps: briefly. (1) The precursor 5,10,15,20-tetrakis(pentafluorophenyl)porphyrin **[H_2_(TPFPP)]** was synthesized by condensation of pyrrole with pentafluorobenzaldehyde in the presence of acetic acid and nitrobenzene under reflux conditions. (2) The tetra-substituted porphyrin 5,10,15,20-tetrakis[(4-carboxyphenyl) thio-2,3,5,6-tetrafluorophenyl] (simplified by **P**) was obtained by structural modification of **[H_2_(TPFPP)]** in the presence of the nucleophile 4-mercaptobenzoic acid and pyridine, using DMF as solvent at room temperature for 24 h (Castro et al., 2015). The compound **P** was characterized by ^1^H and ^19^F NMR (Bruker Avance 300 spectrometer at 300.13 and 282.38 MHz), UV-Vis (Agilent 8453 spectrophotometer), and fluorescence (F4500 – Hitachi spectrofluorometer) spectroscopies. In the fluorescent analysis, the widths of both excitation and emission slits were set at 3.0 nm. Additionally, the quantum yield of singlet oxygen (ϕ_Δ_) was determined from the rate of decay of the ^1^O_2_ phosphorescence at 1270 nm using an Edinburgh F900 instrument consisting of a Rainbow OPO (Quantel Laser-France) 10 Hz, 2 mJ/pulse, which was pumped by a Brilliant NdYAG laser (Quantel Laser-France) and using 5,10,15,20-tetraphenylporphyrin (**TPP**) as standard in DMF (ϕ_Δ_ = 0.65) (Castro et al., 2020). The absorbance of the sample in DMF was adjusted to 0.1 at the excitation wavelength (420 nm).

### Porphyrin Immobilized in Chitosan (P-Chitosan)

Chitosan solution in pH 5.2 at a concentration of 6.6 mg/mL was slowly dripped on a porphyrin solution in acetone (0.83 M) under magnetic stirring. The mixture was stirred (900 rpm) in dark conditions for 72 h at room temperature to acetone evaporation. Then, the homogeneous dispersion of the **P**-chitosan was incorporated into the aqueous phase of the nanoparticle’s preparation process (see below).

### Hybrid Nanoparticle Preparation

The nanostrutured lipid carriers-NLCs (**NP**) coated with chitosan or **P**-chitosan (hybrid nanoparticles, **HNP** or **P-HNP**) were prepared in one step by the emulsification-ultrasonication method, a simple and scalable method (Pivetta et al., 2019). The lipid phase composed of 1.4% (m/v) of Crodamol SS and 0.4% (m/v) of oleic acid was melted at 70°C in a water bath. Thereafter, the aqueous solution of Tween^®^ 80 (1.25% m/v) containing or not 0.24% (m/v) of chitosan, or **P**-chitosan at 70°C was added to the lipid phase. The hot emulsion was sonicated for 10 min (Sonics VCX 750, probe of 13 mm, 40% of amplitude) and then the dispersion was cooled at 25°C.

### Particle Size and Zeta Potential

The diameter by intensity and polydispersity index (PdI) were measured by dynamic light scattering using NanoSize ZS (Malvern^®^) with a scattering angle of 90°. The zeta potential (ZP) was determined by electrophoretic light scattering (ELS) using NanoSize ZS (Malvern^®^). Samples (**NP**, **HNP**, and **P-HNP**) were diluted with deionized water (for size) and KCl solution (for ZP) and analyzed at 25°C.

### Porphyrin Immobilization Efficiency (IE%)

The efficiency of porphyrin immobilization in the hybrid nanoparticles was calculated based on free porphyrin (**P**) amount in the dispersion, an indirect method (Equation 1). For this, 500 μL of the **P-HNP** dispersion was centrifugate at 5000 × *g* in an Amicon filter system from 10 kDa for 10 min. The filtrate containing the free **P** was diluted in DMSO and the absorbance was assessed using a UV-Vis (Agilent 8453 spectrophotometer). The concentration of non-immobilized **P** into nanoparticles dispersion was quantified using the molar extinction coefficient (ε) (127,902 M^–1^ cm^–1^) of **P** in DMSO, previously determined using calibration curves. In the equation below, the total amount of **P** added to the nanoparticle was considered 100%.


(1) $$I\,E\%=\frac{\left({\left[P\right]}_{t\,o\,t\,a\,l}-{\left[P\right]}_{\text{free}}\right)}{{\left[P\right]}_{t\,o\,t\,a\,l}}\times 100$$

[*P*]_*total*_, total concentration of *P*; [*P*]_*free*_, free porphyrin concentration.

### Atomic Force Microscopy

The morphology of the **HNP** was performed in a Shimadzu Scanning Probe Microscope (SPM-9600 model) operating in tapping mode. A probe of silicon (PPP-NCHR) was used with a length of 125 ± 10 μm, a resonance frequency of 204–497 kHz, and a constant force of 10–130 N/m. The **HNP** dispersion was dripped on mica, followed by evaporation for 24 h at room temperature before the analysis.

### Cryogenic Transmission Electron Microscopy

The size and the morphology of coated nanoparticles were determined by cryogenic transmission electron microscopy (Cryo-TEM). The **HNP** and **P-HNP** dispersions were dripped in a grid. After 24 h, dry samples were frozen at −184°C and analyzed in a high-resolution transmission electron microscope FEI TECNAI G^2^ F20 (Thermo Fisher Scientific, United States), operating a beam voltage of 200 KeV. TEM images were analyzed in the ImageJ software (NIH, United States) to determine the size distribution of **HNP** and **P-HNP**.

### Differential Scanning Calorimetry

The crystallinity of nanoparticles and their compounds were analyzed by differential scanning calorimetry (DSC) (Shimadzu DSC-50). The lyophilized samples were hermetically sealed in an aluminum pan and heated in the temperature range of 15–350°C. The heating rate was 10°C/min under nitrogen gas flow (3 kgf/cm^2^). The recrystallization index percentage (% IR) was calculated following Equation 2 (Shah and Pathak, 2010).


(2) $$\%IR=\frac{△\,H_{n\,a\,n\,o\,p\,a\,r\,t\,i\,c\,l\,e}}{△\,H_{b\,u\,l\,k\,l\,i\,p\,i\,d}\;x\,f\,r\,a\,c\,t\,i\,o\,n\,o\,f\,l\,i\,p\,i\,d\,p\,h\,a\,s\,e}\times 100$$

### Thermogravimetric Analysis (TGA)

Thermogravimetric analysis (TGA) was carried out using Shimadzu DSC-50. The previously dried samples were hermetically sealed in an aluminum pan. Then, the samples were heated under an inert atmosphere with a rate of 10°C/min in a temperature range from 30 to 880°C.

### Small-Angle X-Ray Scattering

The equipment was adjusted for a sample detector distance of 3.7 m to investigate sizes around 100 nm. The radiation used was Copper Kα (λ = 1.54 Å) and a measurement range of 0.038 < q < 1.03 (nm^–1^). Scattering data were collected using a two-dimensional Pilatus 300K detector. To guarantee the normalization of the data and the correct subtraction of medium contribution, capillary sample holders glued on steel liners were used. The azimuthal integration of the images was performed with the FIT2D program (Hammersley, 2016). Data processing was carried out according to standard procedures (Oliveira, 2011). The data were normalized to an absolute scale using water as a primary standard. Ten frames of 30 min were obtained for each sample. The treated data were compared to evaluate the stability of the sample and, later, experimental data were optimized. For the analysis, the Indirect Fourier Transformation method (Glatter, 1977) was used on a slightly different implementation (Oliveira et al., 2009). As a result, the theoretical fit of the scattering intensity and the corresponding pair distance distribution function [p(r)] are obtained. The overall shape of the p(r) curve provides indications of the particle shape in the system (Oliveira, 2011).

### Evaluation of Porphyrin Photophysical Properties

Free porphyrin (**P**), **P**-chitosan, and **P-HNP** at 200 nM were evaluated regarding UV-Vis (Agilent 8453 spectrophotometer) and fluorescence (F4500 – Hitachi spectrofluorometer) spectroscopies. **P-HNP** and **P**-chitosan were prepared in deionized water, whereas free porphyrin (**P**) was prepared in DMSO 1% (v/v). The controls of chitosan, **NP**, and **HNP** were prepared in deionized water according to the same volume used for the **P** samples. All samples were excited at 420 nm. In the fluorescent analysis, the widths of both excitation and emission slits were set at 3.0 nm. The absorption and emission spectra were normalized.

### Accelerated Stability

The accelerated stability of the nanoparticles was performed in a Dispersion Analyzer LUMiSizer 6120 centrifuges (L.U.M. GmbH, Berlin, Germany) using the SEPView v.6.4 software. This dispersion analyzer allows simultaneous recording of the intensity of transmitted light (808 nm) in dispersed systems as a function of time and the position of the sample in the cuvette using space and time resolved extinction profiles (STEP-technology) (Caddeo et al., 2013; Tan et al., 2016; Zielinska et al., 2019). **NP**, **HNP**, and **P-HNP** were added in 2 mm disposable polycarbonate sample cells. Measurements were performed at 25°C and the light transmission profile was acquired by the detector every 70 s for 250 min (200 profiles) at a rotation speed of 3801 rpm and light factor of 1. This analysis allows to detect signs of instability such as sedimentation, creaming, coalescence, flocculation, or phase separation (Caddeo et al., 2013; Tan et al., 2016; Zielinska et al., 2019). Instability indexes were calculated by SEPView v.6.4. The parameters used in this experiment mimic the stability or shelf life of 12 months (Caddeo et al., 2013; Tan et al., 2016; Zielinska et al., 2019).

### Mucoadhesion Studies *in vitro*

To assess the mucoadhesive property of the coated nanoparticles *in vitro*, a mucin solution (1 mg/mL) was titrated under the nanoparticle dispersions diluted 50 times. This titration was performed in an MPT-2 accessory of the ZetaSizer Nano ZS. The concentration range analyzed was 0–0.05 mg mL^–1^. For each point of the titration, the ZP of **HNP** and **P-HNP** was determined.

### T24 Bladder Cancer Cells Culture

The human BC cells T24 were obtained from the Rio de Janeiro Cells Bank (BCRJ). T24 cells were cultured in RPMI medium supplemented with 10% of fetal bovine serum and 1% of a streptomycin-penicillin mixture and kept at 5% of CO_2_ and 37°C (MCO-170AIC-PE, PHCbi, Canada).

#### Cytotoxicity (Dark Condition)

The cytotoxicity of free porphyrin (**P**), **HNP**, and **P-HNP** was evaluated by the resazurin assay. T24 cells were seeded in 96-well plates at a density of 2 × 10^4^ cells/well. After 24 h, the cells were treated with different concentrations of the samples (12.5–300 nM) prepared in RPMI phenol red free medium supplemented with 2% of fetal bovine serum and 1% of streptomycin-penicillin mixture (RPMI medium with 2% FBS). **P** stock solutions were prepared in DMSO (1 mM) and then diluted in RPMI medium [(DMSO) <0.5%]. After 6 or 24 h, the cells were washed with 200 μL PBS and then 200 μL of resazurin solution (25 μg/mL) in RPMI medium without phenol red and serum was added to each well. The cells were incubated at 37°C for 4 h. After this period, the fluorescence was then measured with excitation at 530 nm and emission at 590 nm in a microtiter plate reader spectrophotometer (Synergy^TM^ HTX Multi-Mode Microplate Reader, BioTek). DMSO (20% v/v) was used as positive control and untreated cells (cells + RPMI medium) were used as negative control.

#### Photocytotoxicity

Photodynamic studies were carried out in T24 cells with free porphyrin (**P**), **HNP**, and **P-HNP** using the resazurin assay. Briefly, 2 × 10^4^ cells/well were seeded and then incubated at 37°C for 24 h. After incubation, cells were treated with the samples at the same concentrations used in the cytotoxicity experiment. After 6 or 24 h of incubation, the cells were washed with PBS, and then fresh RPMI medium with 2% FBS was added in each well. Immediately, the cells were irradiated at room temperature using a set of LEDs with the emission band ranging between 385 and 425 nm at an irradiance of 24.5 mW cm^–2^ with a total light dose of 5 or 10 J/cm^–2^. The cells were incubated for 20 h at 37°C in a CO_2_ incubator. Then, the resazurin solution was added and the cells were incubated for 4 h. After incubation, fluorescence was evaluated under the same conditions described previously. In the assay with a treatment time of 6 h, the cells were irradiated with only the fluence of 10 J/cm^2^.

The phototoxicity was also evaluated in T24 cells using Cell-titer Glo 2.0 Cell Viability Assay Kit in the same condition described above. After incubation, cells were treated with the samples at the range concentrations (12.5–300 nM) and then incubated. After 6 h, the cells were washed with PBS, and then fresh RPMI medium with 2% FBS was added in each well. The cells were exposed to 10 J/cm^2^ at room temperature. Then, the cells were incubated at 37°C in a CO_2_ incubator for 20 h. After incubation, Cell-titer Glo 2.0 reagent was added to each well according to Promega Protocol, and cells were incubated at room temperature. After 10 min, samples were transferred to 96 wells opaque plates, and luminescence was measured in a microtiter plate reader spectrophotometer (Synergy ^TM^ HTX Multi-Mode Microplate Reader, BioTek). For those studies, the positive and negative controls were the same as those used for cytotoxicity assays. The IC_50_ values were calculated using Prism 5.01 (GraphPad Software Inc., San Diego, CA, United States) software.

### Evaluation of Porphyrin Uptake by Laser Scanning Confocal Microscopy

For the laser scanning confocal microscopy studies, 80,000 cells were placed in each well of a glass-bottom plate (four divisions/wells, CELL view dish-Greiner Bio-One, Brazil). After 24 h, the cells were treated with **P** or **P-HNP** at 10 μM for 30 min. Then, the cells were washed twice with PBS and marked using Fluoroshield with DAPI (nucleus probe, Sigma-Aldrich). After 2 h of incubation, the cells were analyzed by a laser scanning confocal microscope (LEICA-TCS SP2) using a magnification of 64× a diode and HeNe laser with blue filter for DAPI and **P** (λ_excitation_ = 405 nm). Untreated cells (cells + RPMI medium) market with DAPI were used as a negative control. The bandwidth established for emission detection ranged from 411 to 540 nm for DAPI and from 630 to 710 nm for **P**.

### Statistical Analysis

The results of size, PdI, and ZP were analyzed by Prism 5 (GraphPad Software Inc., San Diego, CA, United States) software, using one-way analyses of variance (ANOVA) followed by Tukey. Phototoxicity results were analyzed using two-way analyses of variance followed by Bonferroni post-tests, with a significance level of 5% (0.05), 1% (0.001), and 0.1% (0.001). Data were expressed as mean ± standard deviation (SD).

## Results and Discussion

### Physicochemical Characterization of Nanostructures

This study explored 5,10,15,20-tetrakis[(4-carboxyphenyl) thio-2,3,5,6-tetrafluorophenyl] porphyrin (**P**) as a photosensitizer. It was chosen due to its interesting photophysical properties and encouraging results when immobilized in chitosan film, against *Listeria monocytogenes* biofilm after the light exposition (Castro et al., 2017). This porphyrin has never been applied as a photosensitizer against cancer cells.

Porphyrins have been immobilized or encapsulated in nanostructures as a strategy to avoid the potential problems intrinsic to these compounds, such as aggregation (Zhou et al., 2016). Furthermore, this strategy may increase the delivery of these compounds within the target cells, leading to an interesting intracellular localization to potentialize the effect of this PS on PDT (Lavado et al., 2015; Zhao et al., 2016; Kwiatkowski et al., 2018). Among the nanostructures, chitosan nanoparticles, and lipid nanoparticles have been explored as PS delivery systems (Anderski et al., 2018; Mahlert et al., 2019; Michy et al., 2019; Zhang et al., 2019). However, our group developed solid lipid–polymer hybrid nanoparticles to immobilize porphyrin on its surface, a new particle that has not to date been explored in the PDT area. Lipid–polymer hybrid nanoparticles are a powerful approach to mitigate disadvantages to lipid and polymeric nanoparticles, achieving a nanostructure with superior features (Wong et al., 2006; Wang et al., 2018; Khan et al., 2019).

The polymer chitosan is a non-toxic and biodegradable polysaccharide that plays a special role in porphyrin immobilization due to its ability to form stable complexes with negative charge compounds and substances (Knorr, 1984; Muzzarelli, 1996; Yang et al., 2009; Ferreira et al., 2016; Castro et al., 2017). In the pH of chitosan solution and the hybrid nanoparticles dispersions (pH *ca.* 5.2), the chitosan amino groups (–NH_2_) are protonated (–NH_3_^+^), with high positive density, and may interact with carboxylate *meso*-substituents groups from porphyrin (Knorr, 1984; Muzzarelli, 1996; Khunawattanakul et al., 2008; Yang et al., 2009; Mohammed et al., 2017). This interaction was promoted in two steps: (1) A solution of porphyrin in acetone was mixed with a homogeneous chitosan solution (pH = 5.2) under stirring to immobilize **P** on the chitosan surface by electrostatic interaction; (2) The homogeneous dispersion of **P**-chitosan acetone-free was mixed with an aqueous surfactant solution that then, after heating, was added to the melted lipid phase to obtain NLC coating with porphyrin-chitosan (**P-HNP**). The **NP** showed negative ZP while the hybrid nanoparticle (**HNP** and **P-HNP**) exhibited a positive value (Table 1). This result confirmed the success of the carrier’s coating process with the hypothesis of the chitosan adsorption in **NP** negative surface since this natural polymer is well known for its positive charge (Cheung et al., 2015; Liu et al., 2016a). Furthermore, values of ZP > ∣20 mV∣ could prevent aggregates formation by electrostatic repulsion forces (Feng and Huang, 2001).

**TABLE 1:** Values of size, polydispersity index (PdI), zeta potential (ZP), pH of the formulations **NP**, **HNP**, and percentage of immobilization efficiency (%IE) of **P-HNP**.

Samples	Size (nm)	Size SD	PdI	PdI SD	ZP (mV)	ZP SD	pH	IE%	SD IE%
NP	71.3	3.2	0.122	0.07	–11.3	0.7	3.7	–	–
HNP	100.9	11.1	0.207	0.009	22.5	0.3	5.29	–	–
P-HNP	127.4	4.5	0.249	0.018	27.1	1.2	5.22	95.9	1.2

*IE%, percentage of immobilization efficiency; SD, standard deviation, *n* = 3.*

The nanoparticle surface modification with chitosan and **P**-chitosan significantly increased the hydrodynamic size and changed the ZP of **HNP** and **P-HNP** (*p* < 0.05, one-way ANOVA) (Table 1). Several studies have described the increase of lipid nanoparticles after the chitosan coating process (Liu et al., 2016a; Vieira et al., 2018; Malgarim Cordenonsi et al., 2019). However, the PdI was only significantly influenced by the incorporation of **P**-chitosan (*p* < 0.05, one-way ANOVA) (Table 1). Additionally, PdI values were below 0.3 for all nanoparticles, which is a characteristic of nanoparticles with low polydispersity and a narrow range of size distribution (Üner et al., 2004; Tomasina et al., 2013).

The percentage of immobilization efficiency (IE%) of porphyrin on **HNP** was measured through an indirect method and was high (*ca.* 96%).

The accelerated stability study showed that **NP** presented a low instability index of 0.065 and kept its transmittance profile practically unchanged over the 1-year, being considered stable in this period at a storage temperature of 25°C (Supplementary Figure S2). The hybrid nanoparticles **HNP** and **P-HNP**, over 1 year at 25°C, exhibited a tendency to instability phenomena, with signs of creaming (Supplementary Figure S2), exhibiting instability indexes of 0.818 and 0.700 (Pereira et al., 2018). However, over 31 days, the **P-HPN** was considered stable with instability indexes of 0.105, showing a stability time 1.6-fold higher than **HNP**. Thus, the porphyrin incorporation increased the **HNP’s** long-term stability.

### Atomic Force Microscopy and Transmission Electron Cryo-Microscopy

**FIGURE 1:**
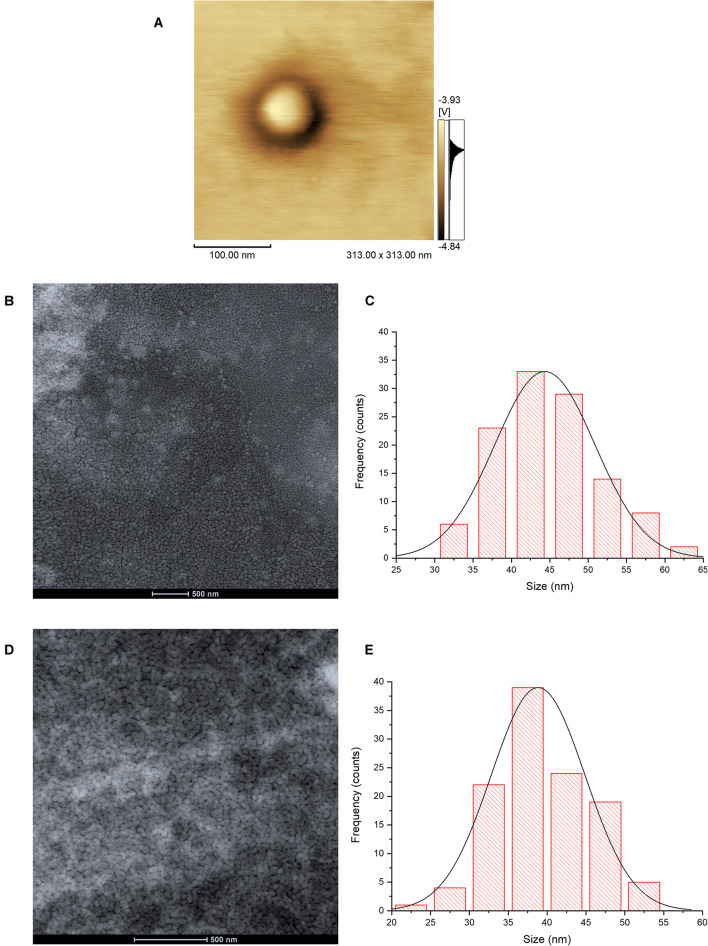
Atomic force microscopy image of **HNP** in phase mode **(A)**; Cryo-TEM images of **HNP (B)** and **P-HNP (D)**; size distribution histograms of **HNP (C)** and **P-HNP (E)**.

The image of atomic force microscopy (AFM) in the phase mode shows a color variation on the particle, suggesting the structure core-shell of **HNP** due to the difference of materials in the surface (Figures 1A,B). The histograms from the Cryo-TEM analysis showed that both **HNP** and **P-HNP** exhibited a homogeneous distribution with well-defined nanoparticles size (Figures 1D,E). The **HNP** size ranged from 30 to 65 nm, whereas the size of **P-HNP** varied between 20 and 55 nm (Figures 1D,E).

### Thermal Analysis: Differential Scanning Calorimetry and Thermogravimetry

The chitosan addition decreased the recrystallization index (RI) of nanoparticles from 27.4 to 25.3%, probably because of the enhancement of lattice defects in the lipid matrix of **NP** triggered by the interaction among chitosan, lipids, and surfactants (Table 2; Vieira et al., 2018; Malgarim Cordenonsi et al., 2019).

**TABLE 2:** Enthalpy, melting point, and recrystallization index (RI) of solid lipid (Crodamol SS), **NP**, **HNP**, and **P-HNP**.

Samples	Enthalpy (J/g)	Melting point (°C)	RI (%)
Crodamol SS	156.3	48.2	100
NP	60.0	47.0	27.4
HNP	55.3	46.1	25.3
P-HNP	62.0	48.6	28.3

Furthermore, the porphyrin incorporation increased the melting point (°C) of the nanoparticles from 46.1 to 48.6°C and the RI from 25.3 to 28.3% when compared with nanoparticles without porphyrin (Table 2).

Both chitosan and **P**-chitosan coating increased the thermal stability of nanoparticles as shown in TGA (Table 3). **HNP** and **P-HNP** showed a remaining weight percentage (RW %) of 37.5 and 40.5%, respectively, for the second maximum degradation temperature (*T*_dmax2_), whereas **NP** had only 20.7% of remaining weight for a lower temperature of 415°C (Table 3). Furthermore, the **HNP** presented a lower weight percentage (*ca.* 4.4% less) than the values obtained for **P-HNP** at *T*_dmax3_, which suggests that the incorporation of **P** improves the **P-HNP** heating resistance.

**TABLE 3:** Thermogravimetric data of the porphyrin (**P**), chitosan (**Ch**), nanostructured particles (**NP**), hybrid nanoparticles (**HNP**), and hybrid nanoparticles with porphyrin (**P-HNP**).

Sample	*T*_di_^a^ (°C)	RW (%)	*T*_dmax1_^b^ (°C)	RW^c^ (%)	*T*_dmax2_ (°C)	RW (%)	*T*_dmax3_ (°C)	RW (%)	*T*_dmax4_ (°C)	^c^ RW (%)
P	290	84.7	336	80.3	400	70.4	456	62.8	622	44.9
Ch	260	87.3	315	73.6	–	–	–	–	–	–
NP	188	96.6	369	47.2	415	20.7	–	–	–	–
HNP	246	96.8	360	68.8	422	37.5	511	10.8	–	–
P-HNP	252	90.5	350	71.0	424	40.5	507	15.2	–	–

*^*a*^*T*_*di*_, initial degradation temperature.*

*^*b*^*T*_*dmax*_, maximum degradation temperature.*

*^*c*^RW%, remained weight percentage.*

### Small-Angle X-Ray Scattering

**FIGURE 2:**
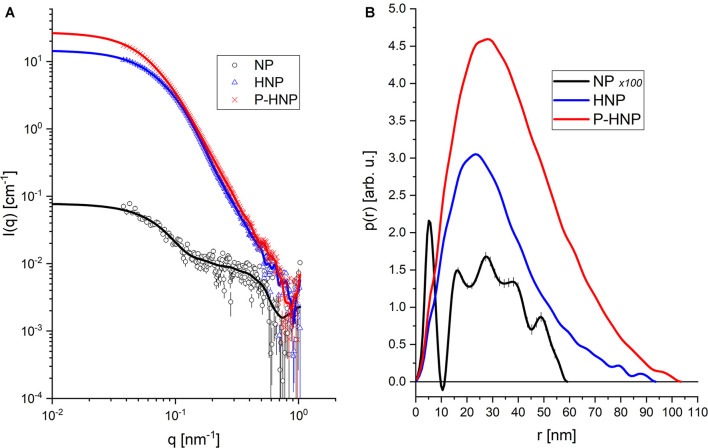
Experimental data from SAXS and Indirect Fourier Transform (IFT) modeling. **(A)** Experimental data (symbols) and theoretical model (solid lines). **(B)** Curves of distance distribution between pairs [p(r)] obtained for each sample.

For a better understanding of the changes in the structure of nanoparticles after chitosan and **P**-chitosan incorporation, X-ray scattering analysis was carried out. The X-ray scattering intensity in the origin, I (0), and radius of gyration (Rg) of **NP**, **HNP**, and **P-HNP** are shown in Figure 2 and Table 4, respectively. Small-angle X-ray scattering (SAXS) intensity varies according to the concentration and size of nanoparticles (Li et al., 2016). Thus, the results suggested that the addition of coatings modulated the size and concentration of the nanoparticles. **HNP** and **P-HNP** exhibited well-defined size and higher concentrations of nanostructures when compared to nanoparticles without chitosan, which can be correlated with the increase in X-ray scattering (Figure 2A). Consequently, nanoparticles prepared with chitosan showed greater efficiency in the self-organization of components. Furthermore, the p(r) function for (**HNP**) or (**P-HNP**) with bell-shape suggests the interaction of the chitosan with other formulation components to form globular (spherical) nanostructures, while non-defined size and morphology were verified for **NP** (Figure 2B). These results agree with the AFM and Cryo-TEM images (Figure 1).

**TABLE 4:** Values of X-ray scattering intensity in the origin [I(0)] and radius of gyration (Rg) of **NP**, **HNP**, and **P-HNP**.

Sample	I(0) (cm^–^^1^)	Rg (nm)
NP	0.076	21.8
HNP	14.64	25.7
P-HNP	27.19	29.9
		

### Photophysical Properties

**FIGURE 3:**
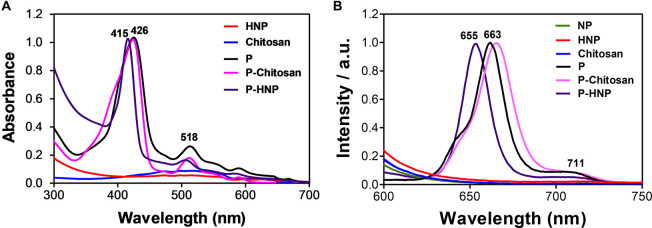
Normalized absorption and emission spectra of free porphyrin (**P**), immobilized porphyrin (**P**-chitosan and **P-HNP**), and controls (**NP**, chitosan, and **HNP**). **(A)** Absorption spectra; **(B)** emission spectra, λ_*exc*_ = 420 nm.

The absorption and emission spectra of the porphyrin are shown in Figure 3. The non-immobilized porphyrin (**P**) showed the typical Soret band at 421 nm attributed to the S_0_ → S_2_ transitions, and four weak Q-bands between 506 and 584 attributed to the S_0_ → S_1_ transitions (Gouterman, 1961). The value of singlet oxygen quantum yield (ϕ_Δ_) found to **P** was 0.69 (Supplementary Figure S3). The UV-Vis spectra of the **P**-chitosan and **P-HNP** materials showed the typical porphyrin absorption profile. Additionally, the spectra of the nanoparticles (**HNP**) and raw chitosan did not present bands in the region of 400 nm (Figure 3A), confirming the successful immobilization.

Castro et al. (2017) and Ferreira et al. (2016) immobilized porphyrins in a chitosan film and observed the broadening and slightly red-shifted in the solid state. The same behavior was observed for **P**-chitosan in solution when compared to the **P** in an aqueous solution of DMSO (1%) at a concentration of 10 μM, red-shifted of Soret (*ca.* 4–5 nm) and Q-bands (*ca.* 4–5 nm). For **P-HNP**, the Soret and Q-bands are blue-shifted (*ca.* 5–11 nm). The emission spectra of **P**-chitosan and **P-HNP** also exhibited two emission peaks with the first fluorescence vibrational mode more remarkable than the second one, slightly red-shifted or blue-shifted effects were observed, respectively, compared to the non-immobilized porphyrin **P** according to the absorption spectra. These shifts are attributed to the small alterations in the porphyrin molecular environment to maximize the interaction with the support. Non-immobilized porphyrin (**P**) displayed two emission peaks at 663 and 711 nm, whereas **P-HNP** exhibited peaks at 655 and 710 nm, upon excitation at 420 nm (Figure 3B). Typical emission features of free or immobilized porphyrin allow cellular uptake studies and subcellular localization. As expected, both the nanoparticles and chitosan in absence of porphyrin **P** are non-luminescent. Furthermore, these results confirm that the process of nanoparticle preparation did not interfere with the photophysical properties of **P** to act as PS.

### Mucoadhesion Evaluation *in vitro*

In general, when the drugs are administered in the bladder in an intravesical way, it shows a short residence time with easy elimination by the periodic emptying bladder, which decreases the therapeutic effect (Weintraub et al., 2014). A strong interaction of drugs or nanoparticles with the mucous bladder layer (the most superficial layer) may increase the residence time of the drug. Thus, the dose and number of administrations might be lower, and the biological effect improved (Weintraub et al., 2014). Chitosan can extend the residence time of nanoparticles in mucous membranes (Fonte et al., 2011; Liu et al., 2016b; Anderski et al., 2018). Additionally, this natural polymer was described as absorption-promoting due to its capacity of open tight junctions (Artursson et al., 1994; Sonaje et al., 2012). Hence, nanoparticles coated with chitosan are an interesting strategy to improve the permanence of nanoparticles in the bladder (Erdoğar et al., 2012).

**FIGURE 4:**
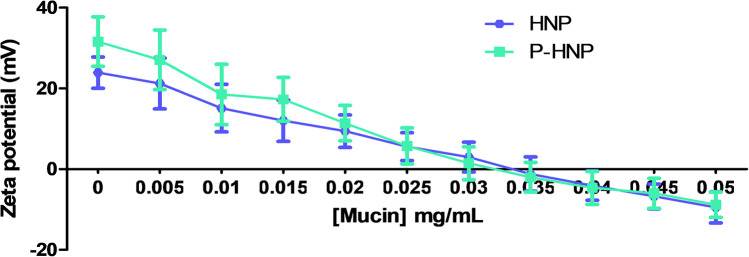
Zeta potential values of **HNP** and **P-HNP** as function of mucin concentration in the mucoadhesion assays.

The main expected mechanism accountable for the mucoadhesive property of nanocarriers is the electrostatic interaction (Yoncheva et al., 2011; Bhatta et al., 2012). The *in vitro* mucoadhesion studies employed by us explored the ionic interaction between the positive charge surface of hybrid nanoparticles and the negative sialic groups of mucin. This ionic interaction can be observed by the change in the ZP value of the nanoparticle as shown in Figure 4. A decrease in the positive charge of **HNP** and **P-HNP** by mucin concentration increase can be observed, indicating a mucoadhesive property of these particles (Figure 4). At 0.4 mg/mL of mucin, the **HNP** and **P-HNP** exhibited a negative ZP. Rençber et al. (2016) showed that polymeric nanoparticles coated with chitosan also displayed a reduction in the ZP after incubation with 0.1% of mucin.

### Cytotoxicity and Phototoxicity Assays

**FIGURE 5:**
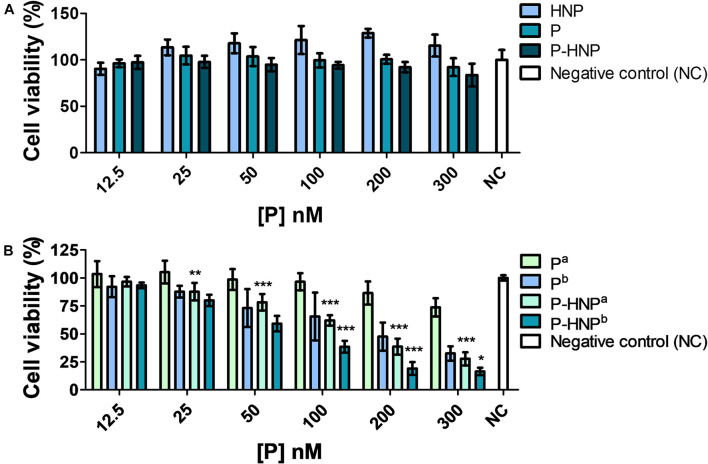
Cell viability of T24 assessed by resazurin. **(A)** Cytotoxicity of hybrid nanoparticles (**HNP**), free porphyrin (**P**), and immobilized porphyrin on hybrid nanoparticles (**P-HNP**) after 24 h of treatment in the dark conditions. **(B)** Phototoxicity of free porphyrin (**P**) and porphyrin immobilized on hybrid nanoparticles (**P-HNP**) after 24 h of treatment followed by laser irradiation. ^*a*^5 and ^*b*^10 J/cm^2^ dose of light. Negative control (NC)-untreated cells (cells + RPMI medium). The results are presented as mean ± SD of three independent experiments performed in triplicate. Significant differences relative to **P** and **P-HNP**, in each photoinduction dose, are indicated with an asterisk. Statistical significance: ***p < 0.001, **p < 0.01, *p < 0.05. Data obtained by two-way ANOVA analysis followed by Bonferroni post-test.

The study in the dark condition revealed that **HNP**, **P-HNP**, and free **P** were considered no cytotoxic for T24 cells once the results showed cell viability above 76% for all concentrations evaluated (Figure 5A; ISO, 2009). These data are relevant because the property of PS to be non-toxic under dark is crucial to its application in clinical trials (Nawalany et al., 2009).

The experiments of PDT using 24 h of treatment showed that **P-HNP** and **P** displayed a light dose-dependency phototoxicity (Figure 5B), while **HNP** did not show phototoxicity against T24 cells for both fluences assessed (Supplementary Figure S4). **P**, as well as **P-HNP**, were phototoxic to T24 cells for concentrations above 50 nM. In the concentration of 300 nM, **P** reduced the cell viability to 32.5%, whereas **P-HNP** was reduced to 16.3%. The IC_50_ for **P-HNP** was 2.2-fold smaller for cells exposed to 10 J/cm^2^ (IC_50_ = 66.5 nM) than for the cells exposed to 5 J/cm^2^ (IC_50_ = 149.5). Comparing the fluences of 5 and 10 J/cm^2^, the IC_50_ of **P-HNP** were, respectively, 3.2- and 2.5-fold lower than free **P** (IC_50_ = 483.7 and 165.8 nM). Thus, **P-HNP** exhibited superior phototoxicity than **P**.

**FIGURE 6:**
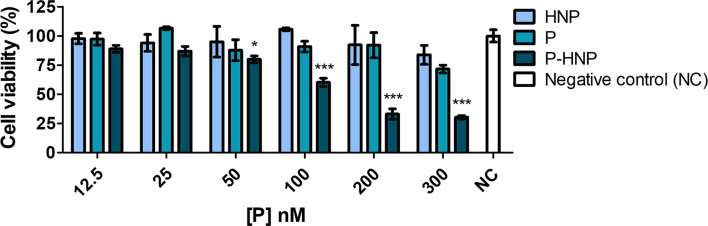
Phototoxicity of free porphyrin (**P**) and porphyrin immobilized on hybrid nanoparticles (**P-HNP**) in T24 cells evaluated by resazurin after 6 h of treatment followed by photoinduction with a dose of 10 J/cm^2^. Negative control (NC)-untreated cells (cells + RPMI medium). The results are presented as mean ± SD of three independent experiments performed in triplicate. Significant differences relative to **P** and **P-HNP**, in each photoinduction dose, are indicated with an asterisk. Statistical significance: ***p < 0.001, *p < 0.05. Data obtained by two-way ANOVA analysis followed by Bonferroni post-test.

A phototoxicity assay using reduced treatment time (6 h) at a fluence of 10 J/cm^2^ was also performed. This study suggested that 6 h of treatment is enough for **P-HNP** delivery of **P** into cells and improves their phototoxicity. For the concentrations of 300, 200, and 100 nM, **P-HNP** significantly reduced the cell viability when compared to free **P** (Figure 6). The **P-HNP** IC_50_ was 130.6 nM, whereas the IC_50_ of free **P** could not be estimated because it was non-toxic for cells.

Furthermore, a bioluminescence ATP assay was carried out to evaluate the free **P** and **P-HNP** phototoxicity against T24 BC cells. This assay was chosen because it is considered more sensitive, robust, and precise than colorimetric and fluorescence assays. It also has a high correlation between ATP detection and cell viability values, since ATP production is immediately interrupted during cell death (Maehara et al., 1987; Zumpe et al., 2010).

**FIGURE 7:**
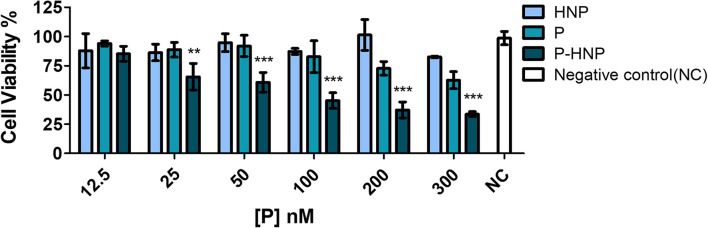
Phototoxicity of free porphyrin (**P**) and porphyrin immobilized on hybrid nanoparticles (**P-HNP**) in T24 cells using ATP bioluminescent assay after treatment of 6 h followed by PDT with 10 J/cm^2^ of fluence. Negative control (NC)-untreated cells (cells + RPMI medium). The results are presented as mean ± SD (*n* = 3). Significant differences relative to free **P** and **P-HNP** are indicated with an asterisk. Statistical significance: ***p < 0.001, **p < 0.01. Data obtained by two-way ANOVA analysis with post-tests of Bonferroni.

The same phototoxicity profile observed in resazurin assay for **P** and **P-HNP** were also verified in the ATP experiment, confirming the significant phototoxic effect of **P-HNP** (Figure 7). As result, **P-HNP** exhibited significantly higher phototoxic effects when compared with free **P** for all concentrations except to 12.5 nM. The IC_50_ of **P-HNP** was 86.12 nM, while the free **P** was non-toxic for cells in the concentration range evaluated.

The superior activity of **P-HNP** compared to free **P** can be attributed to the favorable physical and chemical properties of hybrid nanoparticles, which promoted a quick and efficient **P** delivery into cells, avoiding **P** aggregation. In this context, we evaluated the photophysical properties of **PS** in the cell culture medium under dark conditions. The free porphyrin (**P**) exhibited a broad Soret band when compared to free **P** in DMSO 1% (Supplementary Figure S5A). **P** self-aggregation changed drastically its absorption spectral profile, once the bands enlarged and decreased the absorption intensity as shown in Supplementary Figure S4. Consequently, the emission intensity decreased, and new bands appeared. Such changes may be due to the mixture of monomeric and aggregates species of porphyrin that trigger fluorescence quenching (Supplementary Figure S5B). However, when assessed in cell culture medium, **P** immobilized in nanoparticles (**P-HNP**) was stable, and its aggregation was minimized since the absorption and emission spectra of the PS were similar to those shown in Figure 3.

### Evaluation of Porphyrin Uptake by Laser Scanning Confocal Microscopy

**FIGURE 8:**
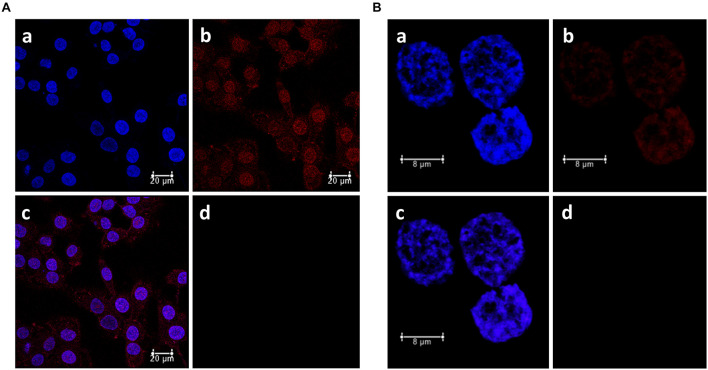
Subcellular localization of porphyrin (**P**) in bladder cancer cells T24 determined by confocal laser scanning microscopy. **(A) P-HNP** uptake. **(B)** Free **P** uptake. **a:** DAPI (blue fluorescence), **b:** porphyrin (red fluorescence), **c:** merged, **d:** control (untreated cells + DAPI, emission 630–710 nm). T24 cells were incubated sequentially with **P** or **P-HNP** for 30 min and marked with DAPI (nucleus probe).

Figure 8A showed the high uptake of immobilized **P** (**P-HNP**) into T24 cells for only 30 min of incubation (red fluorescence). This high and fast porphyrin internalization explains the enhancement of the phototoxicity of **P-HNP** previously described (see Figures 5–7). The use of nanoparticles as a PS delivery system increased the accumulation of **P** at strategic points in the cancer cell. In contrast, the free **P**, in the same conditions of incubation, exhibited a weaker fluorescence when compared with **P-HNP** that may be due to the free aggregation phenomena in cell culture medium (Figure 8B). According to Nawalany et al. (2009) and Brezaniova et al. (2016), the high internalization and accumulation of PS are responsible for the great phototoxic effect of porphyrin.

Photosensitizer is supposed to be in specific organelles from cells to trigger the mechanism involved in cellular death by the production of ROS including singlet oxygen (Nawalany et al., 2009; Bacellar et al., 2015; Gazzi et al., 2019). The merged fluorescence images showed that **P-HNP** is distributed throughout the cell, including the nucleus (Figures 8A,B). Nevertheless, free **P** was localized only in the nucleus. Some studies describe that the DNA can also be oxidized in PDT treatments (Bacellar et al., 2015). This is a strategy explored in cancer treatment (Zhu et al., 2017; Kadhim et al., 2019). Porphyrin and metalloporphyrins can break DNA single-strand by photoinduction mechanisms with singlet oxygen action or by cleavage of the sugar moieties from nucleic acids (Defedericis et al., 2006). Anti-tumor drugs into nuclei can inhibit the replication and transcription of DNA, wrecking genetic material and inducing cells to apoptosis (Wang et al., 2011). Therefore, targeting-nuclei drugs are considered interesting and efficient to kill cancer cells (Zhu et al., 2017). However, nucleus and organelle dual-targeting drugs have been applied successfully in cancer therapy once the drugs can act trigger two different mechanisms, increasing drug efficiency on tumors as reported by Zhu et al. (2017). Thus, those results suggest that the dual-targeting and the reduced aggregation showed by the immobilized **P** (**P-HNP**) can explain the higher phototoxic effect of this PS compared to free **P** in BC cells.

## Conclusion

The unique properties of the hybrid nanoparticles developed in this study increased the stability, improved the photophysical properties of the porphyrin in the cell culture medium, and quickly and efficiently delivered it within T24 cells. The application of **P-HNP** triggered a potent phototoxic effect (low IC_50_ = 66.5 nM) in PDT against T24 BC cells. Thus, this study represents an advance to the development of macrocycle incorporation in biodegradable hybrid nanoparticles as a possibility to potentialize their PDT action. Furthermore, the hybrid nanoparticles presented mucoadhesive properties in studies *in vitro*. This property is interesting for BC treatment since it might improve the permanence time of nanoparticles in the bladder after its intravesical administration. When these results are together, the porphyrin delivery system developed in this study has the potential to be applied in other cancer models and should be explored in animal models with the aim of enhancing PDT.

## Data Availability Statement

The original contributions presented in the study are included in the article/Supplementary Material, further inquiries can be directed to the corresponding author.

## Author Contributions

LS: porphyrin synthesis, development of nanoparticles, materials preparation, and biological studies, and writing—original draft. KC: porphyrin synthesis and characterization, and writing—review and editing. CB: biological studies, review, and editing. RS: writing—review and editing and supervision. CO: writing, review, and editing. PM: validation, writing—review and editing, and supervision. All authors contributed to worte the manuscript and approved the final version of the manuscript.

## Conflict of Interest

The authors declare that the research was conducted in the absence of any commercial or financial relationships that could be construed as a potential conflict of interest.

## Publisher’s Note

All claims expressed in this article are solely those of the authors and do not necessarily represent those of their affiliated organizations, or those of the publisher, the editors and the reviewers. Any product that may be evaluated in this article, or claim that may be made by its manufacturer, is not guaranteed or endorsed by the publisher.
