# Supplementary material for: Hybrid Nanoparticles as an Efficient Porphyrin Delivery System for Cancer Cells to Enhance Photodynamic Therapy
Source: Front Bioeng Biotechnol. 2021 Sep 17;9:679128. doi: 10.3389/fbioe.2021.679128 (PMC8484888; doi:10.3389/fbioe.2021.679128)
Supplement: Supplementary file 1 [file Data_Sheet_1.pdf]

Supplementary Material

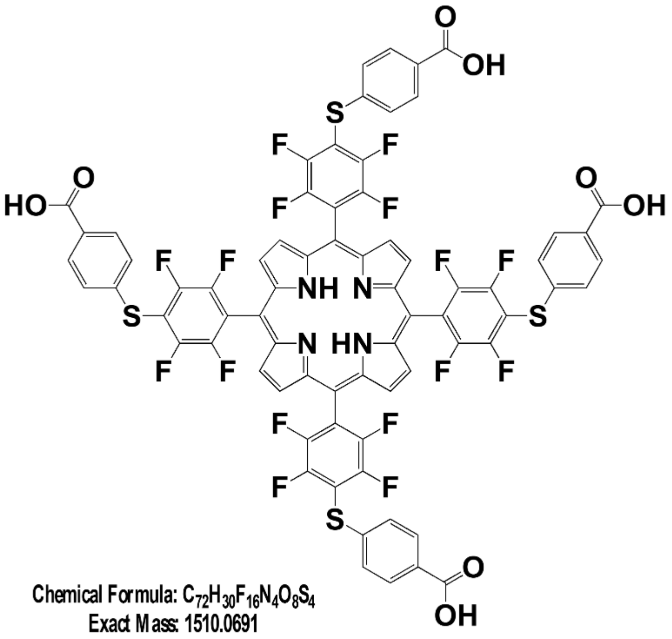

Supplementary Figure 1. Chemical Structure of 5,10,15,20-tetrakis [(4- carboxyphenyl) thio-2,3,5,6-tetrafluorophenyl] porphyrin **(P).**

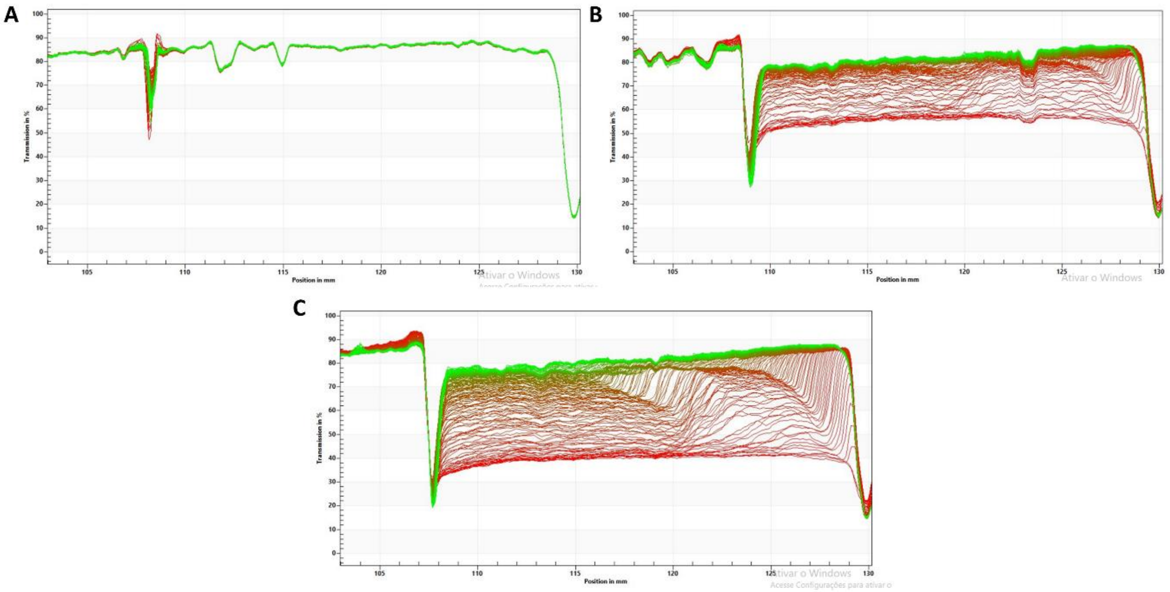

Supplementary Figure 2. Normalized NIR transmission profiles versus samples cells position for **(A)** nanostructured lipid carrier (**NP**), (**B**) hybrid nanoparticles (**HNP**), **(C) P** immobilized in hybrid nanoparticles (**P-HNP**) by analytical centrifugation in LUMiSizer® for 250 min at 3801 rpm at 25°C.

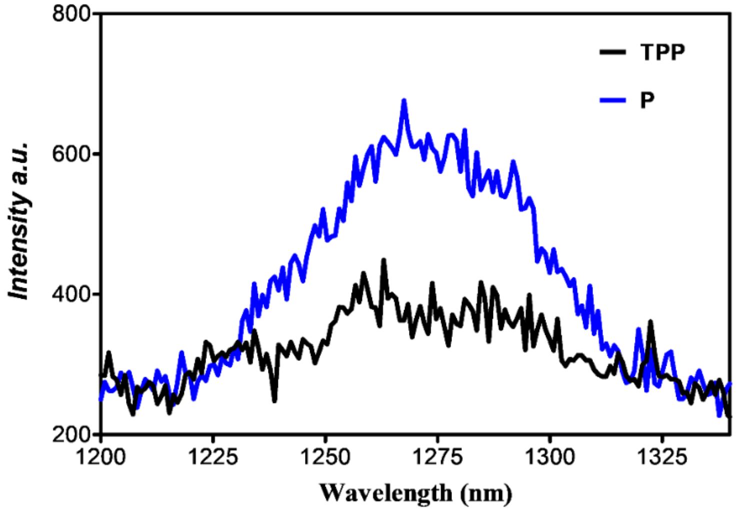

Supplementary Figure 3. Fluorescence emission of singlet oxygen ($^1O_2$) for free porphyrin (**P**) and 10,15,20-tetraphenylporphyrin (**TPP**) (standard) in DMF.

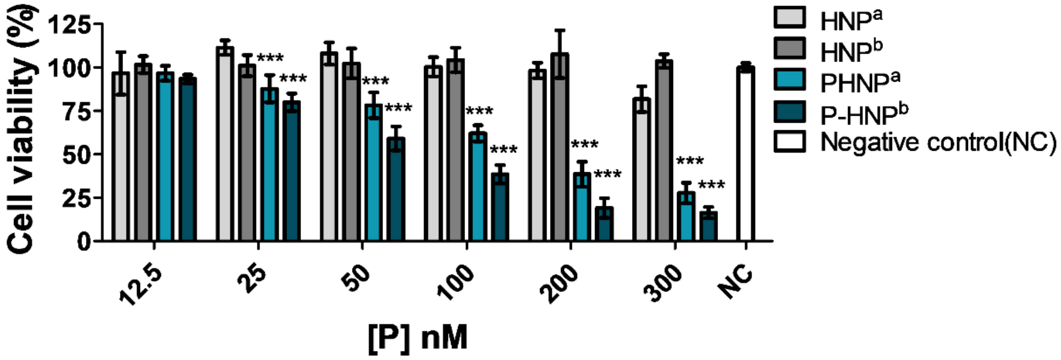

Supplementary Figure 4. Phototoxicity of hybrid nanoparticles (**HNP**) and porphyrin immobilized on hybrid nanoparticles (**P-HNP**) after 24 h of treatment followed by laser irradiation. [a]5 J/cm^2 dose of light and [b]10 J/cm^2 dose of light. Negative control (NC)-untreated cells (cells+Medium). The results are presented as mean ± standard deviation of three independent experiments performed in triplicate. Significant differences relative to **HNP** and **P-HNP**, in each photoinduction dose, are indicated with an *. Statistical significance: ***p < 0.001. Data obtained by Two-way ANOVA analysis followed by Bonferroni post-test.

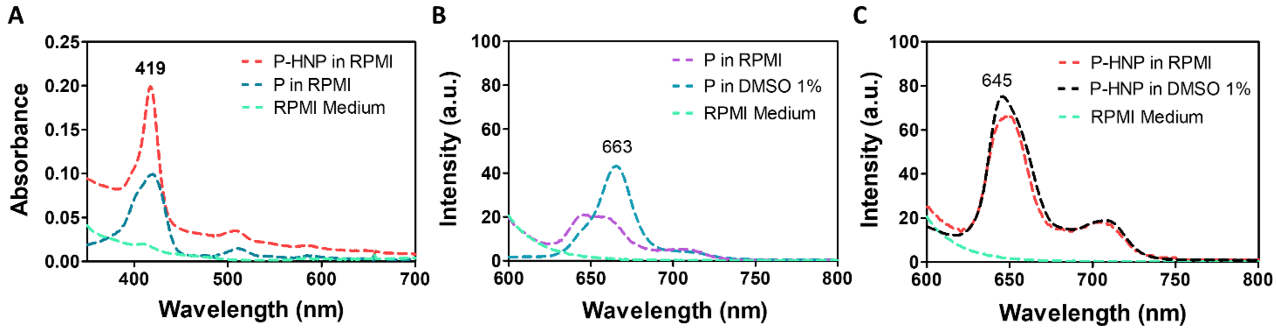

Supplementary Figure 5. (A) Absorption spectra of non-immobilized porphyrin (**P**), immobilized porphyrin (**P-HNP**) and controls (RPMI medium with 2% fetal bovine serum). **(B)** Emission spectra of **P** in RPMI medium, **P** in aqueous solution of DMSO 1% and control of RPMI. **(C)** Emission spectra of **P-HNP** in RPMI medium, **P-HNP** in DMSO solution (1%) and control of RPMI (Emission spectra, λ_{exc} = 420 nm). All solutions were prepared in RPMI medium with 2% fetal bovine serum.
