# Supplementary figures and images for: Hybrid Nanoparticles as an Efficient Porphyrin Delivery System for Cancer Cells to Enhance Photodynamic Therapy
Source: Front Bioeng Biotechnol. 2021 Sep 17;9:679128. doi: 10.3389/fbioe.2021.679128 (PMC8484888; doi:10.3389/fbioe.2021.679128)

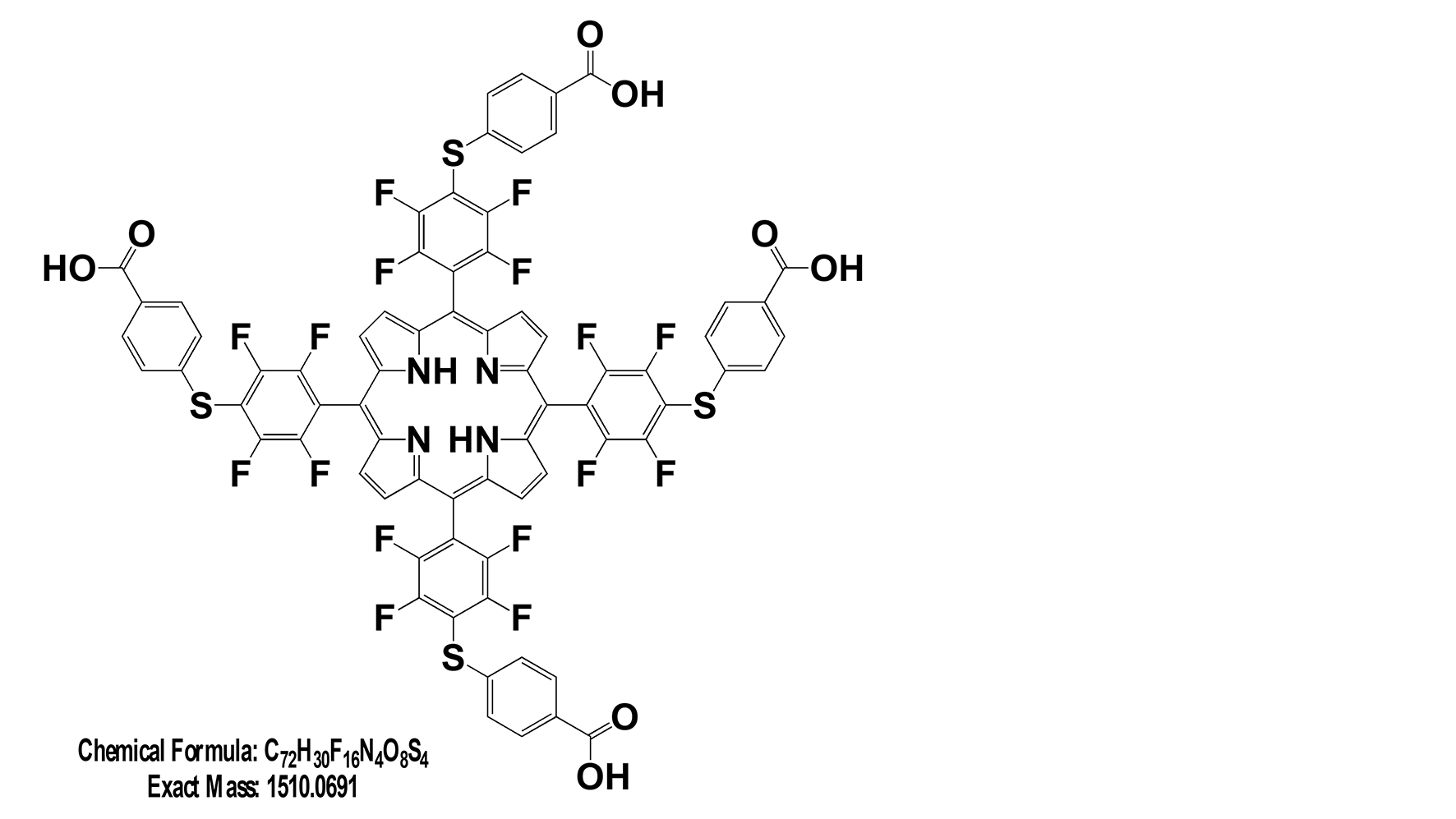

Supplement: Supplementary file 2 [file Image_1.TIF]

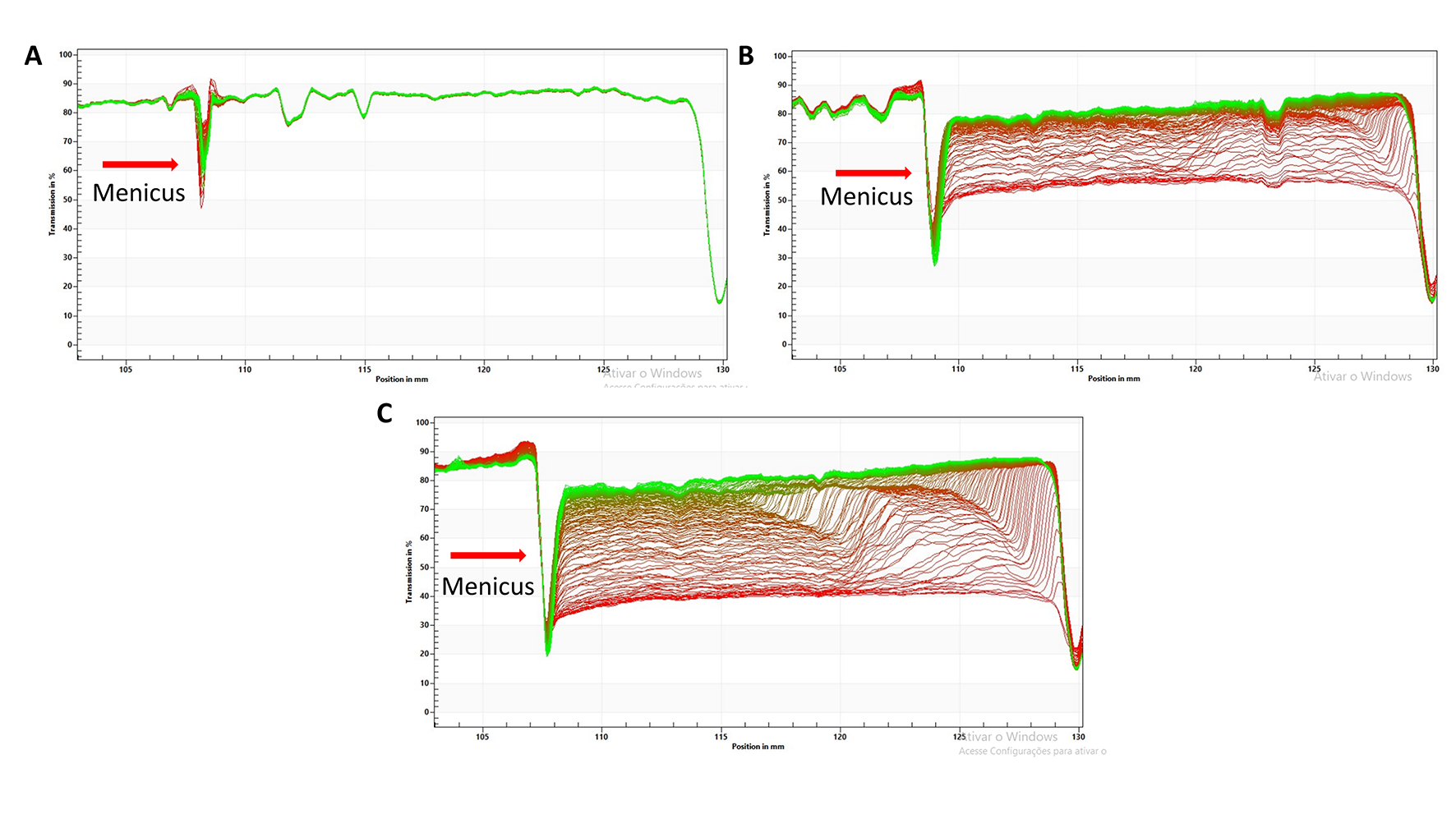

Supplement: Supplementary file 3 [file Image_2.TIF]

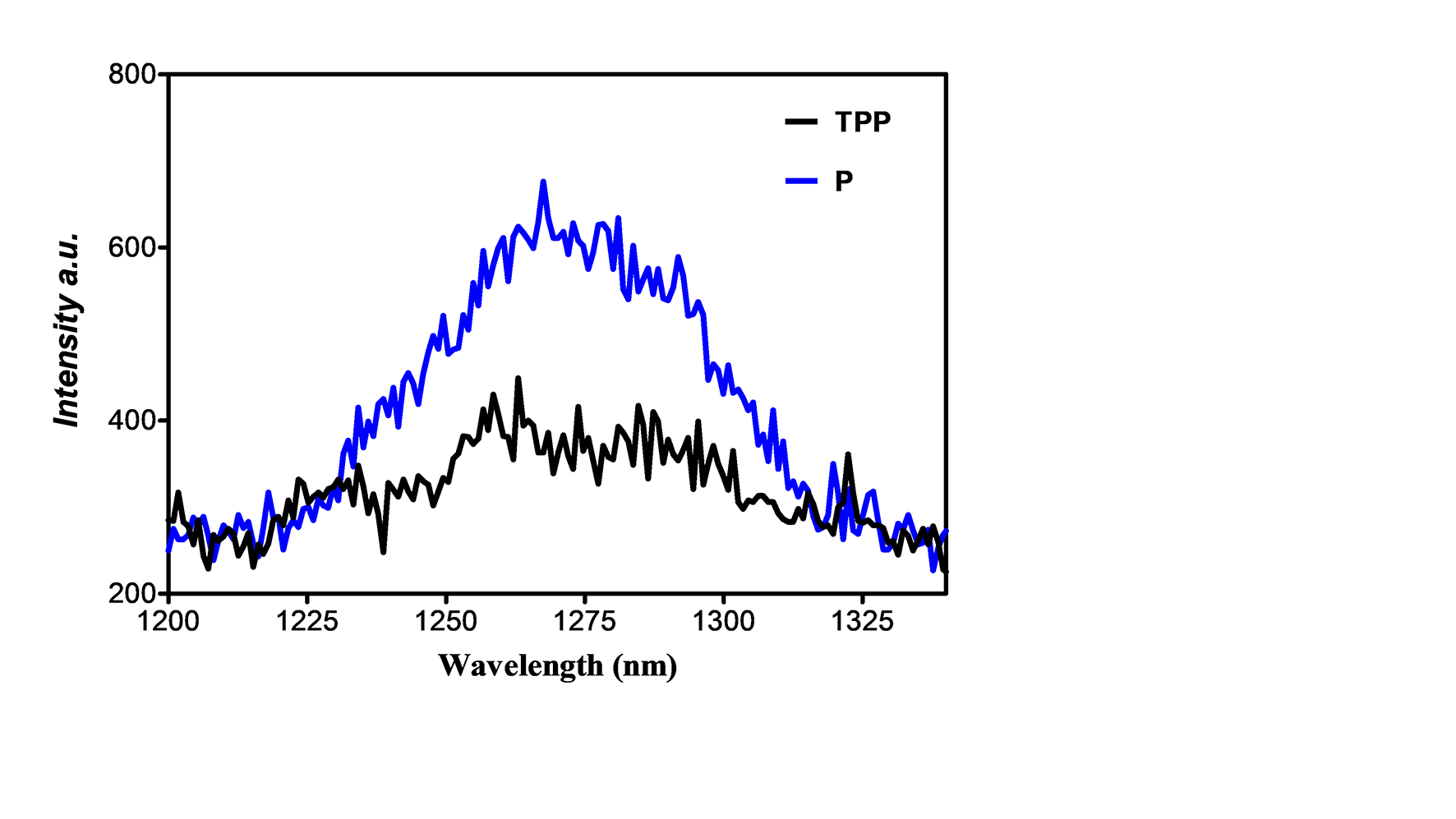

Supplement: Supplementary file 4 [file Image_3.TIF]

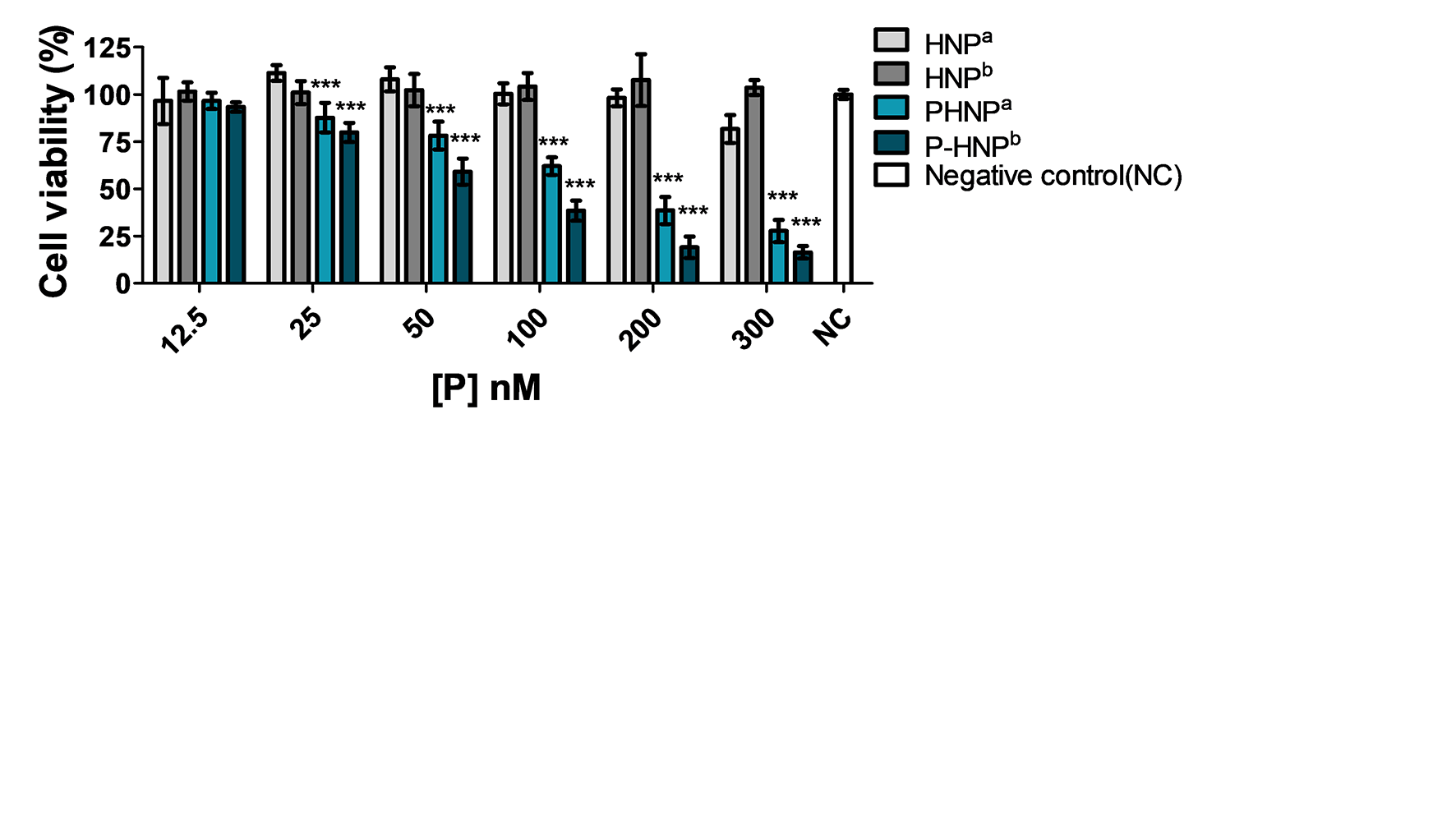

Supplement: Supplementary file 5 [file Image_4.TIF]

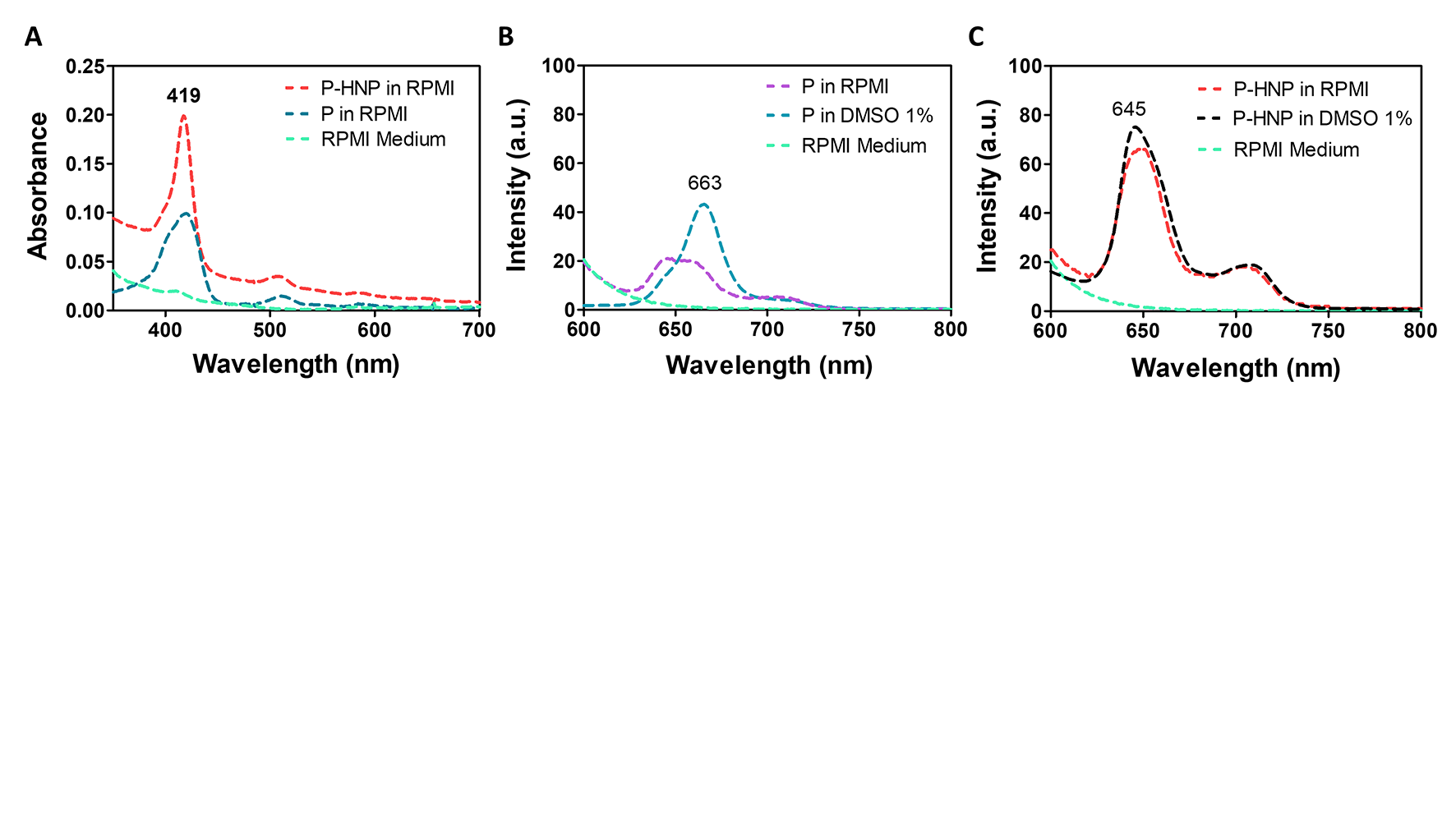

Supplement: Supplementary file 6 [file Image_5.TIF]
